# A recombinant scFv antibody-based fusion protein that targets EGFR associated with IMPDH2 downregulation and its drug conjugate show therapeutic efficacy against esophageal cancer

**DOI:** 10.1080/10717544.2022.2063454

**Published:** 2022-04-13

**Authors:** Shiming He, Chunyan Zhao, Hongyu Tao, Weijin Sheng, Ruijuan Gao, Xiujun Liu, Yongsu Zhen

**Affiliations:** Institute of Medicinal Biotechnology, Chinese Academy of Medical Sciences, Peking Union Medical College, Beijing, China

**Keywords:** Esophageal squamous cell carcinoma, esophageal cancer, antibody-drug conjugate, Fv-LDP-D3-AE

## Abstract

The present study aimed to evaluate the anti-tumor efficacy of the epidermal growth factor receptor (EGFR)-targeting recombinant fusion protein Fv-LDP-D3 and its antibody-drug conjugate Fv-LDP-D3-AE against esophageal cancer. Fv-LDP-D3, consisting of the fragment variable (Fv) of an anti-EGFR antibody, the apoprotein of lidamycin (LDP), and the third domain of human serum albumin (D3), exhibited a high binding affinity for EGFR-overexpressing esophageal cancer cells, inhibited EGFR phosphorylation and down-regulated inosine monophosphate dehydrogenase type II (IMPDH2) expression. Fv-LDP-D3 was taken up by cancer cells through intensive macropinocytosis; it inhibited the proliferation and induced the apoptosis of esophageal cancer cells. *In vivo* imaging revealed that Fv-LDP-D3 displayed specific tumor-site accumulation and a long-lasting retention over a 26-day period. Furthermore, Fv-LDP-D3-AE, a pertinent antibody-drug conjugate prepared by integrating the enediyne chromophore of lidamycin into the Fv-LDP-D3 molecule, displayed highly potent cytotoxicity, inhibited migration and invasion, induced apoptosis and DNA damage, arrested cells at G2/M phase, and caused mitochondrial damage in esophageal cancer cells. More importantly, both of Fv-LDP-D3 and Fv-LDP-D3-AE markedly inhibited the growth of esophageal cancer xenografts in athymic mice at well tolerated doses. The present results indicate that Fv-LDP-D3, and Fv-LDP-D3-AE exert prominent antitumor efficacy associated with targeting EGFR, suggesting their potential as promising candidates for targeted therapy against esophageal cancer.

## 1. Introduction

Esophageal cancer (EC) is one of the most common cancer worldwide with a high mortality and a relatively low overall 5-year survival rate. EC is divided into two pathological subtypes, namely esophageal squamous cell carcinoma (ESCC) and esophageal adenocarcinoma (EAC) (Pennathur et al., 2013; Fatehi Hassanabad et al., 2020; He et al., 2021). In the Chinese population, ESCC is the main EC subtype and has a high incidence (Pennathur et al., 2013; Lu et al., 2021). EAC is less common in China, while being more prevalent in western countries, and has a short median survival time (Fujihara et al., 2017). Currently, chemotherapy represents the main treatment for EC, but it is associated with considerable toxicity (Liu et al., 2019). In recent years, targeted therapy has shown promising anti-tumor efficacy. EGFR is a member of the ERBB (the erythroblastic leukemia viral oncogene homolog) receptor tyrosine kinase family of transmembrane receptor proteins, which include an extracellular ligand-binding domain, a transmembrane domain, and an intracellular kinase domain (Ciardiello & Tortora, 2003). EGFR is overexpressed in most esophageal tumors (Hanawa et al., 2006). In general, EGFR overexpression is more common in ESCC than in EAC (Kawaguchi et al., 2007). Cetuximab, an EGFR inhibitor monoclonal antibody, has a significant therapeutic effect against EGFR-overexpressing ESCC (Zhu et al., 2018). Gong et al. used pingyangmycin and cetuximab to treat EC xenograft in athymic mice, reporting an enhanced therapeutic efficacy under combination treatment as opposed to monotherapy (Gong et al., 2012). In addition, the combination of cetuximab and trastuzumab has shown a synergistic anti-tumor effect against EC both *in vitro* and *in vivo* (Yamazaki et al., 2012).

In general, antibody-drug conjugates (ADC) consist of three parts, that is, the antibody, a linker, and a small-molecule cytotoxic drug. The antibody facilitates targeting, the linker connects the antibody to the small-molecule cytotoxic drug, and the latter exerts cytotoxicity against tumor cells. Hu et al. previously prepared an EGFR-targeting ADC (LR004-VC-MMAE), which exhibited favorable anti-tumor activity in mouse models of EC (Hu et al., 2019). Therefore, EGFR represents a promising target for the treatment of EC.

Human serum albumin (HSA) can be internalized into cancer cells, where its degradation contributes to the supply of free amino acids, representing a major energy source for tumors (Davidson et al., 2017). This altered state of extracellular protein metabolism can be exploited for the targeted delivery of anti-tumor drugs. HSA domain III (D3)-modified single-chain variable fragment (scFv) have an extended serum half-life while retaining their specific binding efficacy (Andersen et al., 2013). Targeted delivery of anti-tumor drugs via albumin has shown anti-tumor potential in previous studies (Kratz, 2014; Shan et al., 2018). Furthermore, IMPDH2 is a rate-limiting enzyme in the *de novo* biosynthesis of guanine nucleotides. It is overexpressed in various types of cancers, and its upregulation is related to poor prognosis, promoting tumor formation and development (Ying et al., 2018; Kofuji et al., 2019; Sahu et al., 2019). It has been suggested that IMPDH2 can be used not only as a biomarker for tumor diagnosis, but also as a potential therapeutic target for the treatment of malignant tumors. However, rarely studies have been reported on IMPDH2 as a potential therapeutic target for esophageal cancer.

Lidamycin (LDM, also known as C-1027) is an anti-tumor antibiotic currently undergoing phase II clinical trial. LDM is composed of an active enediyne chromophore (AE), with extremely potent cytotoxicity, and a non-covalently bound apoprotein LDP. LDP can bind to AE and stabilize the enediyne structure through hydrophobic interactions. In particular, AE and LDP can be isolated and reconstituted *in vitro* (Shao & Zhen, 1995; Tanaka et al., 2001; Shao & Zhen, 2008). Thus, LDM can be used as an effective “warhead” agent for the construction of antibody-drug conjugate through a unique process, DNA recombination, and molecular reconstitution.

ScFv with good penetration and distribution in tumor tissues has been used for the targeted delivery of anti-tumor drugs (Trebing et al., 2014). In previous studies, we constructed a novel recombinant fusion protein (Fv-LDP-D3) and its antibody-drug conjugate (Fv-LDP-D3-AE) (Wang et al., 2018). In that recombinant fusion protein (Fv-LDP-D3), Fv is an anti-EGFR single-chain variable fragment, LDP is the apoprotein of LDM, and D3 is the domain III of HSA; Furthermore, the antibody drug conjugate (Fv-LDP-D3-AE) was prepared by integrating the active enediyne chromophore AE of LDM into the fusion protein. The study demonstrated the efficacy of the antibody drug conjugate (Fv-LDP-D3-AE) against K-Ras-mutated pancreatic cancer (Wang et al., 2018).

However, whether Fv-LDP-D3 and Fv-LDP-D3-AE have anti-cancer activity against EC and their underlying molecular mechanism has not yet been studied. In this study, we evaluated the anti-tumor activity of Fv-LDP-D3 and Fv-LDP-D3-AE in EC *in vitro* and *in vivo*, exploring their potential mechanisms of action. Based on the current findings, the constructed recombinant fusion protein and the antibody-drug conjugate may be potential candidates for EGFR-targeted EC therapy.

## 2. Materials and methods

### 2.1. Reagents

The Annexin V FITC Apoptosis Detection Kit, Cell Cycle Assay Kit-PI/RNase Staining Kit, and Fluorescein Labeling Kit-NH_2_ were all purchased from DOGINDO (Japan). The DyLight680 Antibody Labeling Kit and BCA protein assay kit were purchased from Thermo Fisher Scientific (Waltham, MA, USA). The Cell Counting Kit-8 was purchased from NCM Biotech (Suzhou, China). Immobilon western chemilum HRP substrate was purchased from Millipore (Burlington, MA, USA). Matrigel^®^ Basement Membrane Matrix was purchased from Corning (Glendale, AZ, USA).

Various antibodies respectively against EGFR (#4267), phospho-EGFR (#3777), PARP (#9542), cleaved PARP (#5625), Caspase-3 (#9662), Cleaved Caspase-3 (#9661), Bcl-2 (#2872), and Bax (#2772), all purchased from Cell Signaling Technology (Danvers, MA, USA). The antibody against IMPDH2 (ab131158) was purchased from Abcam (Cambridge, MA, USA). Antibodies against GAPDH (60004-1-Ig) and β-actin (66009-1-Ig) were purchased from Proteintech (Rosemount, IL, USA), while those against p-Histone H2A.X (sc-517348) and Chk1 (sc-8408) were purchased from Santa Cruz Biotechnology (Santa Cruz, CA, USA).

### 2.2. Cell culture

Human ESCC cell lines KYSE150, KYSE520 were purchased from Creative Bioarray, Inc. Eca109 cell line was provided by our laboratory. KYSE150, KYSE520, and Eca109 cell lines were cultured in Roswell Park Memorial Institute (RPMI) 1640 medium supplemented with 10% fetal bovine serum (FBS) and 1% penicillin-streptomycin. Mouse embryonic fibroblasts cell line NIH3T3 was purchased from the Cell Center of Peking Union Medical College (Beijing, China) and cultured in Dulbecco's modified Eagle medium (DMEM) supplemented with 10% FBS and 1% penicillin-streptomycin. All cells were cultured in a 37 °C, 5% CO_2_ cell incubator.

### 2.3. Binding affinity of Fv-LDP-D3 in vitro

#### 2.3.1. Enzyme-linked immunosorbent assay (ELISA)

KYSE150, KYSE520, NIH3T3 cells were seeded in 96-well plates at 2 × 10^4^ cells per well, placed in a 37 °C, 5% CO_2_ incubator, and cultured for 24 h. After fixing with 4% paraformaldehyde and blocking with 5% skim milk PBS solution, different concentrations of Fv-LDP-D3 were added to the experimental wells and incubated for 2 h. An anti-His tag monoclonal antibody was then added, and the plate was incubated at 37 °C for 1 h, followed by the addition of horseradish peroxidase-conjugated goat anti-mouse IgG and incubation for 40 min. After each incubation, 96-well plates were washed with PBST or PBS. Next, 3,3′,5,5′-tetramethylbenzidine was added, followed by 2 M H_2_SO_4_ to stop the reaction. The absorbance of reaction wells was measured at OD_450_ nm using a microplate reader (Thermo Fisher Scientific, Franklin, MA, USA).

#### 2.3.2. Immunofluorescence

KYSE150 and KYSE520 cells were seeded on 24-well plates at 1 × 10^5^ cells per well and cultured in the incubator for 24 h. Cells were fixed with 4% paraformaldehyde, followed by three washes with PBS. Fv-LDP-D3 (400 ng/mL) was then added to the experimental wells of 24-well plates, followed by incubation for 2 h at room temperature and washing with PBS three times for 3 min each. The anti-His tag monoclonal antibody was added, incubated at 37 °C for 2 h, and washed with PBS three times, 3 min each. The Alexa Fluor^®^ 488-conjugated goat anti-mouse IgG was then added, and the cells were incubated in the incubator for 1 h and washed thrice with PBS. After adding anti-fluorescence attenuation sealing agent (including DAPI), cells were observed and photographed under a fluorescence microscope (200×).

### 2.4. Internalization of Fv-LDP-D3

KYSE150 and Eca109 cells were seeded in an 8-well chamber slide and cultured for 24 h. After adding fluorescein-labeled Fv-LDP-D3 recombinant fusion protein (0.2 mg/mL) alone or in combination with macropinocytosis inhibitor EIPA (80 μM), cells were incubated in the dark and then washed with PBS thrice. The cells were fixed with 4% paraformaldehyde for 0.5 h and washed thrice with PBS for 4 min each time. Anti-fluorescence attenuation sealing agent (including 4′,6-diamidino-2-phenylindole, DAPI) was then added to avoid light reaction at room temperature for 15 min. The cells were then observed and photographed under a fluorescence microscope.

### 2.5. Apoptosis and cell cycle arrest assays

The pro-apoptotic effect of Fv-LDP-D3 and Fv-LDP-D3-AE on KYSE150 and Eca109 cells as well as the effects of Fv-LDP-D3-AE on cell cycle arrest were analyzed via flow cytometry. For apoptosis analysis, the cells were seeded in a 6-well plate at a density of 2 × 10^5^/well and cultured in a 37 °C cell incubator for 24 h. Different concentrations of Fv-LDP-D3 and Fv-LDP-D3-AE were then respectively added to the wells, and the untreated cells were used as controls. Cells were collected according to the instructions of the Annexin V FITC Apoptosis Detection Kit (Dojindo). For cell cycle arrest analysis, cells were seeded at the same density and then collected as per the instructions of the Cell Cycle Assay Kit-PI/RNase staining (Dojindo). Apoptosis and cell cycle arrest was detected via flow cytometry.

### 2.6. In vitro cytotoxicity assay

The cytotoxicity of Fv-LDP-D3 and Fv-LDP-D3-AE was analyzed via the Cell Counting kit-8 method. Briefly, several EC cell lines were seeded in 96-well plates at 3 × 10^3^ cells/well and placed in a 37 °C incubator for 24 h. The cells were then treated with either Fv-LDP-D3 (1 mg/mL) or different concentrations of Fv-LDP-D3-AE. After incubation for 24 h, 10 μL CCK8 reagent were added and incubated for 1 h. Absorbance was measured at OD_450_ nm using a microplate reader, and untreated cells were used as control. Cell survival rate (%) was calculated as per the following formula: [(A sample-A blank)/(A control-A blank)] × 100%. The IC_50_ values were calculated using SPSS software.

### 2.7. Western blot

Briefly, cells were lysed on ice using RIPA tissue/cell lysis buffer for approximately 30 min, and the protein concentration was quantified using the BCA protein assay kit. Equal amounts of protein samples were transferred to PVDF membranes after SDS-PAGE electrophoresis. After blocking, the membranes were incubated respectively with specific antibodies overnight and were subsequently incubated with the secondary HRP-anti-rabbit IgG or HRP-anti-mouse IgG antibodies. Protein bands were visualized with Immobilon Western Chemiluminescent HRP Substrate reagent and captured using a FlourChem E imaging system.

### 2.8. 5‑Ethynyl‑2'‑deoxyuridine (EdU) assay

The experimental procedure was performed according to the EdU kit instructions (Beyotime, China). Briefly, KYSE150 and Eca109 cells were inoculated in an 8-well chamber slide at 37 °C for 24 h. Fv-LDP-D3-AE (12.5 ng/mL) was then added and cultured for 24 h. Then EdU staining buffer was added, and cells were fixed with 4% polyformaldehyde. The nuclei were stained with Hoechst 33342. Cells were observed under a fluorescence microscope and photographed.

### 2.9. Migration and invasion assay

Transwell experiments were performed to evaluate cell migration and invasion. For the migration assay, KYSE150 and Eca-109 cell suspensions of 1 × 10^5^ cells in serum-free cell culture medium were added to the upper wells of transwell chambers (Corning, USA), while 500 μL cell culture medium containing 20% fetal bovine serum was added to the lower wells. Different concentrations of Fv-LDP-D3-AE were added to the upper wells, then the plates were cultured in a cell incubator for 24 h. Cells were fixed with 4% paraformaldehyde, stained with 0.1% crystal violet, a cotton swab was used to gently scrape cells off the inner chamber, and then observed and photographed under an inverted microscope (100×). 33% acetic acid was added for decolorization, and the OD_570_ value was determined using a microplate reader. For the invasion assay, 45 μL of diluted matrigel were added to the transwell chamber and placed in a 37 °C incubator for 2 h to solidify. The rest of the experiments were performed following the experimental procedures described for the migration assay.

### 2.10. Transmission electron microscopy

After treating KYSE150 and Eca-109 cells with Fv-LDP-D3-AE for 24 h, 2.5% glutaraldehyde was added to fix the cells, then 1% osmium tetroxide was added, followed by dehydration under different concentrations of alcohol and 100% acetone. After uranyl acetate lead circuit staining, the cells were observed and photographed using transmission electron microscope (TEM).

### 2.11. In vivo fluorescence imaging assay

*In vivo* fluorescence imaging experiments were performed using KYSE150 and KYSE520 nude mouse xenograft models. The Fv-LDP-D3 recombinant fusion protein was labeled as per the DyLight 680 Antibody Labeling Kit instructions. When tumor sizes reached approximately 180–400 mm^3^, Fv-LDP-D3 was injected intravenously into mice at a dose of 20 mg/kg. Mice were anesthetized with 2% isoflurane, and the *in vivo* distribution of the fusion protein was evaluated using the IVIS-Imaging System (Xenogen). In addition, after the experiment, the mice were sacrificed by the CO_2_ method, and the tumor tissues and main organs of the nude mice were taken out for *in vitro* fluorescence imaging. Imaging signals were analyzed using Living Image software (Xenogen, Alameda, CA, USA).

### 2.12. In vivo therapeutic efficacy

#### 2.12.1. Effect of FV-LDP-D3-AE in the KYSE150 xenograft model

Female BALB/c-*nu* mice (18–22 g), were purchased from SPF (Beijing) Biotechnology Co., Ltd. KYSE150 cells (5 × 10^6^ cells/200 μL) suspended in PBS were inoculated subcutaneously into the right armpit of athymic mice. When the size of tumors reached 400–500 mm^3^, the tumor mass blocks were removed from nude mice and subjected to dissection in sterile saline. Tumor tissue fragments (2 mm^3^) were then transplanted into the right armpit of nude mice with a trocar, and the wound was sealed with celloidin. When the tumor volume reached about 80 mm^3^, nude mice were randomized into two groups (*n* = 6, per group), a control group and a Fv-LDP-D3-AE (0.2 mg/kg) group. The drug treatment group was given by tail vein injection once a week for two consecutive weeks. During the experiment, the long and short diameters of the tumor were measured every 3 days, and the mice were weighed. The tumor volume was calculated as per the following formula: V = AB^2^/2, where A is the longest diameter of the tumor, and B is the shortest diameter perpendicular to A. The tumor growth inhibition rate was calculated as: [1-(terminal tumor volume in the administration group - initial tumor volume in the administration group)/(terminal tumor volume in the control group - initial tumor volume in the control group)] × 100%.

#### 2.12.2. Anti-tumor efficacy of Fv-LDP-D3 and its antibody–drug conjugate FV-LDP-D3-AE in the KYSE520 xenograft tumor model

KYSE520 (3 × 10^6^/0.2 mL) cells were subcutaneously inoculated into the armpit of female BALB/c nude mice (body weight 18–20 g). When the average tumor volume reached about 100 mm^3^, nude mice were divided into groups according to tumor volume and body weight, with five mice per group. Those groups included a control group, Fv-LDP-D3 (20 mg/kg) group, Fv-LDP-D3 (40 mg/kg) group, Fv-LDP-D3-AE (0.25 mg/kg) group, and Fv- LDP-D3-AE (0.5 mg/kg) group. As for the administration schedule, Fv-LDP-D3 (20 mg/kg) and Fv-LDP-D3 (40 mg/kg) were administered twice a week, while Fv-LDP-D3-AE (0.25 mg/kg) and Fv-LDP-D3-AE (0.5 mg/kg) were administered once a week. Treatment continued for two weeks, and administration was done via tail vein injection. The mice were euthanized at the end of after the experimental period. Tumors and various organs were taken and fixed in 10% formalin for hematoxylin and eosin staining. In addition, the expression level of Ki-67 in tumor tissues was detected via immunohistochemistry.

### 2.13. Statistical analysis

Statistical analyses were performed using SPSS 17.0 (SPSS Inc., Chicago, IL) and GRAPHPAD PRISM 6 software (GraphPad Software, Inc., San Diego, CA, USA). All data are presented as the mean ± standard deviation. Statistical differences between groups were determined via the Student's t-test or two-way ANOVA analysis. *p* < .05 was considered to indicate significance. (*p < .05; **p < .01, ***p < .001).

## 3. Results

### 3.1. Binding of Fv-LDP-D3 to EC cells

**Figure 1.**
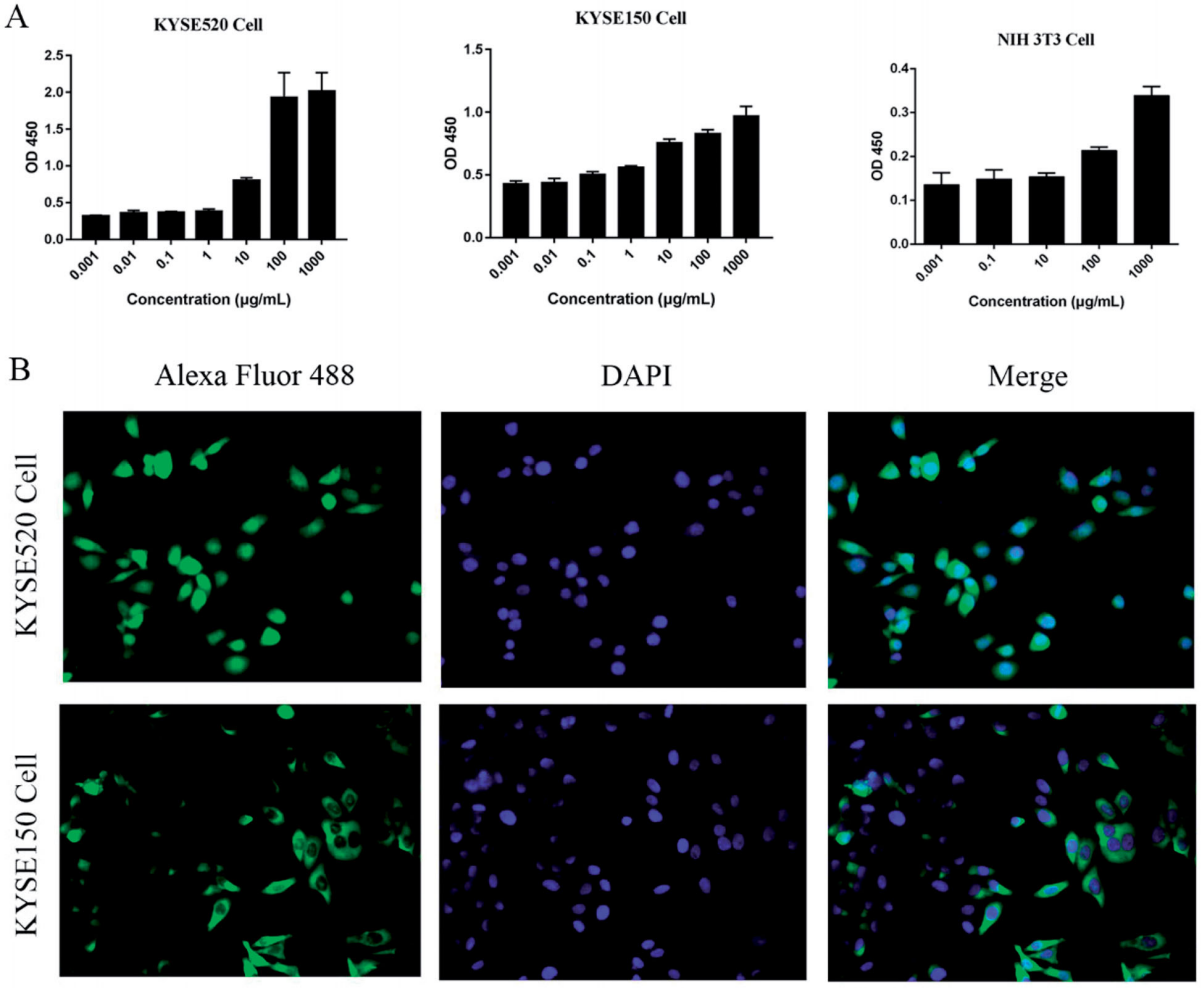
Binding capability of Fv-LDP-D3 to esophageal cancer cells *in vitro*. (A) Binding of Fv-LDP-D3 to KYSE520 cells, KYSE150 cells, NIH3T3 cells respectively, as determined by ELISA. (B) Immunofluorescence analysis of Fv-LDP-D3 binding to KYSE520 and KYSE150 cells (Photo multiple, ×200).

The binding ability of Fv-LDP-D3 was evaluated via ELISA and immunofluorescence analysis. ELISA-based binding assays indicated that the Fv-LDP-D3 recombinant fusion protein could bind to KYSE520, KYSE150 cells respectively in a concentration-dependent manner. However, a very weak binding ability of Fv-LDP-D3 to mouse embryonic fibroblasts cell line NIH3T3 was observed. (Figure 1(A)). Furthermore, KYSE520 and KYSE150 cells exhibited green fluorescence in the cell membrane and cytoplasm, indicative of Fv-LDP-D3 binding to EC cells *in vitro* (Figure 1(B)).

### 3.2. Macropinocytosis-mediated uptake of Fv-LDP-D3 in EC cells

**Figure 2.**
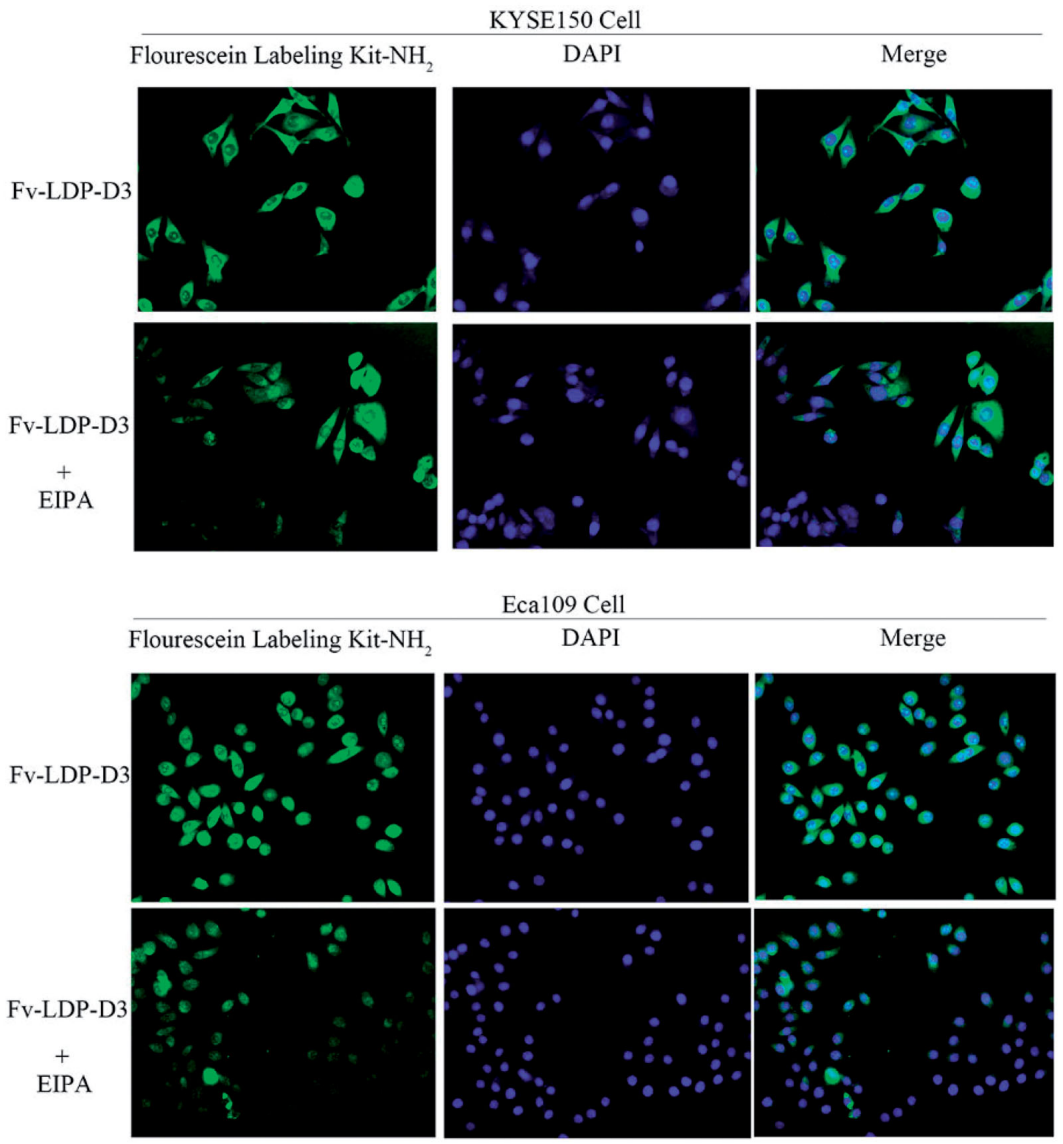
Fv-LDP-D3 uptake by esophageal cancer cells. Fluorescence microscopy observation of the macropinocytosis-mediated uptake of fluorescein-labeled Fv-LDP-D3 in KYSE150 and Eca109 cells treated with or without 80 μM EIPA, respectively (Photo multiple, ×200).

The uptake of Fv-LDP-D3 in EC cells was detected under fluorescence microscope. The recombinant fusion protein Fv-LDP-D3 was labeled with fluorescein, and the nuclei were stained with DAPI. As shown in Figure 2, green fluorescence was observed around DAPI-stained nuclei, indicating that KYSE150 cells and Eca109 cells can uptake the Fv-LDP-D3 protein. Upon addition of EIPA, a specific inhibitor of macropinocytosis, the green fluorescence intensity around nuclei weakened, thus indicating that Fv-LDP-D3 protein was passively taken up by EC cells via macropinocytosis.

### 3.3. Inhibition of EC cell proliferation by Fv-LDP-D3 and Fv-LDP-D3-AE

**Figure 3.**
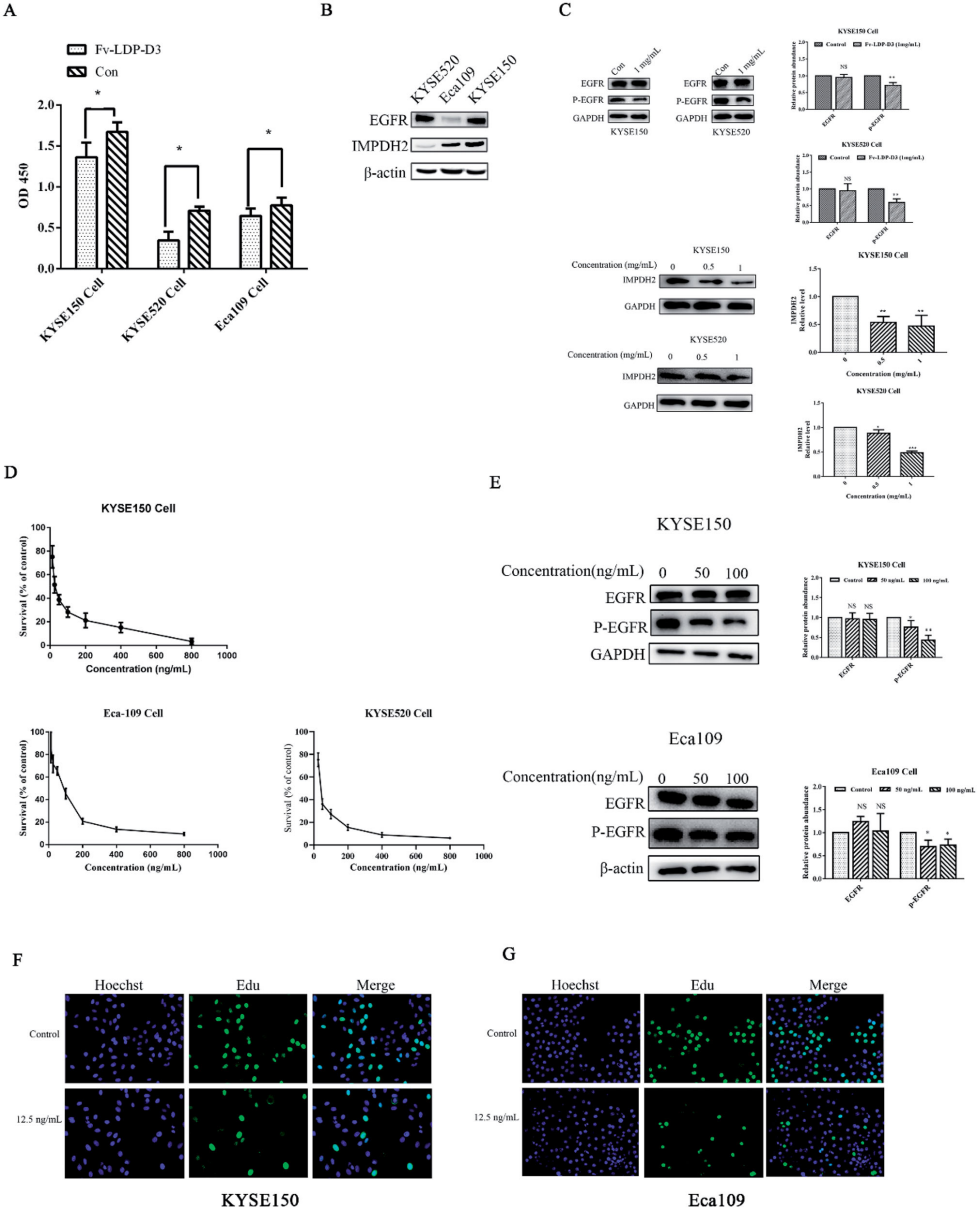
Analysis of Fv-LDP-D3 and Fv-LDP-D3-AE bioactivity. (A) Fv-LDP-D3-induced inhibition of KYSE150, KYSE520, and Eca109 cell proliferation, as determined by CCK8 assay. (B) EGFR and IMPDH2 expression in different esophageal cancer cell lines were determined by western blot analysis. (C) Western blot analysis of EGFR and IMPDH2 expression in KYSE150 and KYSE520 cells treated with Fv-LDP-D3 for 24 h. (D) The cytotoxicity effects of Fv-LDP-D3-AE in different esophageal cancer cell lines were evaluated by CCK8 assays. (E) KYSE150 and Eca109 cells were treated with different concentrations of Fv-LDP-D3-AE for 24 h. Western blot analysis of EGFR and phosphor-EGFR expression. (F,G) The effect of Fv-LDP-D3-AE on the proliferation of KYSE150 and Eca109 cells was determined by EdU staining assays (Photo multiple, ×200). Results are reported as means ± standard deviation (SD) (*n* = 3). NS: no significance, *p < 0.05, **p < 0.01, ***p < 0.001 versus control group.

The CCK8 method was used to evaluate the cytotoxicity of Fv-LDP-D3 and Fv-LDP-D3-AE in KYSE150, KYSE520, and Eca109 cells. As shown in Figure 3(A), when compared to the control group, Fv-LDP-D3 significantly inhibited the growth and proliferation of EC cells (*p < .05). In order to characterize the molecular mechanism of Fv-LDP-D3-related cell growth suppression, we first detected the expression levels of EGFR and IMPDH2 in different EC cells. The highest EGFR expression was detected in KYSE520 cells, and the highest IMPDH2 expression was detected in KYSE150 cells (Figure 3(B)). As shown in Figure 3(C), Fv-LDP-D3 inhibited EGFR phosphorylation and reduced IMPDH2 expression. In all tested cell lines, Fv-LDP-D3-AE exhibited strong cytotoxicity (Figure 3(D); Table 1). In KYSE150 cells, the phosphorylation of EGFR was downregulated, with their downregulation being more pronounced under higher concentrations (Figure 3(E)). In Eca109 cells, Fv-LDP-D3-AE also inhibited phosphorylation of EGFR (Figure 3(E)). These findings indicated that the anti-proliferation effect of Fv-LDP-D3-AE may be mediated via the suppression of EGFR phosphorylation . Cell proliferation was further analyzed via EdU staining. A low proportion of EdU-positive cells was observed in the presence of Fv-LDP-D3-AE as opposed to among control cells, further suggesting that Fv-LDP-D3-AE inhibited cancer cell proliferation (Figure 3(F–G)).

**Table 1.** IC50 values of Fv-LDP-D3-AE to various EC cell lines.

Cell line	IC_50_ (ng/mL)
KYSE150	33 ± 2
Eca109	75 ± 4
KYSE520	44 ± 3

### 3.4. Inhibition of EC cell migration and invasion by Fv-LDP-D3-AE

**Figure 4.**
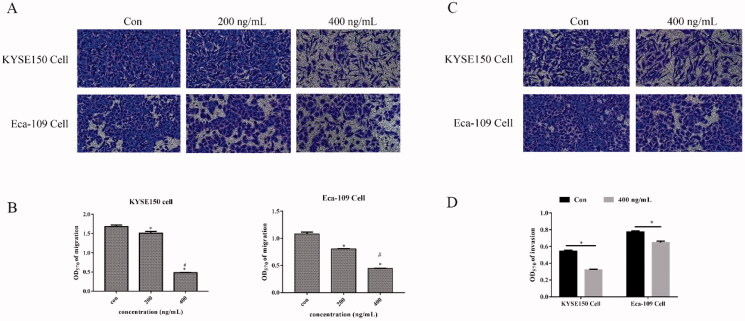
Fv-LDP-D3-AE suppressed EC cell migration and invasion. Transwell assays were performed to assess the effect of Fv-LDP-D3-AE on the migration (A) and invasion (C) of KYSE150 and Eca109 cells, respectively. (B,D) Quantification of cell migration and invasion. *p < 0.05 versus control group, #*p* < 0.05 versus 200 ng/mL.

We evaluated the effect of Fv-LDP-D3-AE on the migration and invasion ability of KYSE150 and Eca109 cells via transwell assays. The experimental results are shown in Figure 4(A,B). Fv-LDP-D3-AE inhibited the migration of EC cells in a concentration-dependent manner. We added matrigel to the transwell chamber to assess the influence of Fv-LDP-D3-AE on EC cell invasion. Fv-LDP-D3-AE inhibited the invasion of EC cells compared to the control treatment (Figure 4(C,D)).

### 3.5. DNA damage, cell cycle arrest, and apoptosis of treated EC cells

**Figure 5.**
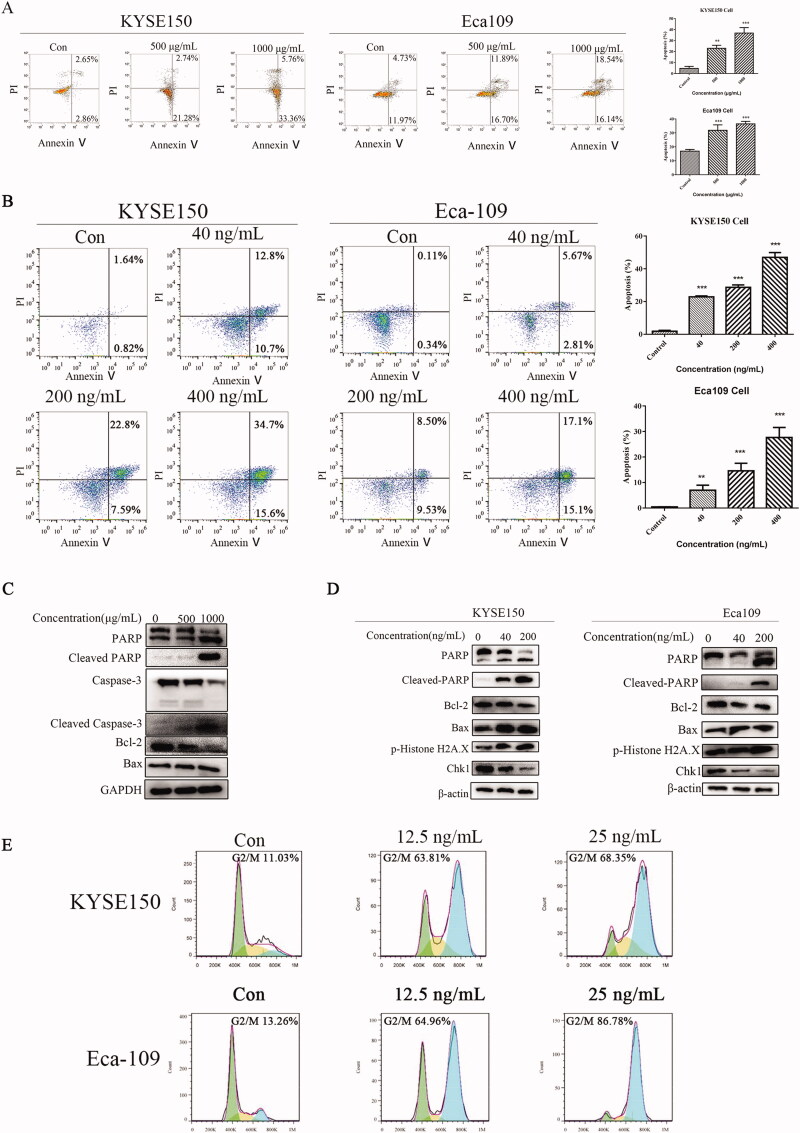
DNA damage, cell cycle arrest analysis, and apoptosis of EC cells. (A,B) Flow cytometry analyses of apoptosis in KYSE150 and Eca109 cells treated with Fv-LDP-D3 and Fv-LDP-D3-AE, respectively. (C) Western blot analysis of apoptosis-related proteins in KYSE150 cells treated with Fv-LDP-D3. (D) Western blot analysis of apoptosis- and DNA damage-related protein expression in KYSE150 and Eca109 cells treated with Fv-LDP-D3-AE. (E) Cell cycle arrest in KYSE150 and Eca109 cells treated with Fv-LDP-D3-AE was determined by flow cytometry. Results are reported as means ± standard deviation (SD) (*n* = 3). **p < 0.01, ***p < 0.001 versus control group.

To determine the pro-apoptotic effect of Fv-LDP-D3 and Fv-LDP-D3-AE on EC cells, KYSE150 and Eca109 cells were incubated with different concentrations of the experimental formulations for 24 h. Higher drug concentrations induced greater apoptosis, with late apoptosis in particular being induced in a concentration-dependent manner (Figure 5(A,B)). Western blot experiments were performed to determine the effects of Fv-LDP-D3 and Fv-LDP-D3-AE on apoptosis-related protein expression. Fv-LDP-D3 suppressed caspase 3 and Bcl-2 expression in a concentration-dependent manner, while cleaved caspase 3 levels increased. At 1 mg/mL, Fv-LDP-D3 suppressed PARP expression, while upregulating cleaved PARP levels, as compared to control treatment (Figure 5(C)). Fv-LDP-D3-AE also upregulated cleaved PARP and down-regulated PARP expression, while suppressing the expression of anti-apoptotic Bcl-2. At 200 ng/mL, Fv-LDP-D3-AE significantly upregulated DNA damage-related p-Histone H2A.X, while suppressing Chk1 (checkpoint kinase 1) levels in a concentration-dependent manner (Figure 5(D)). Further, Fv-LDP-D3-AE induced G2/M phase arrest, once again in a concentration-dependent manner (Figure 5(E)).

### 3.6. Induction of ultrastructural changes by Fv-LDP-D3-AE in EC cells

**Figure 6.**
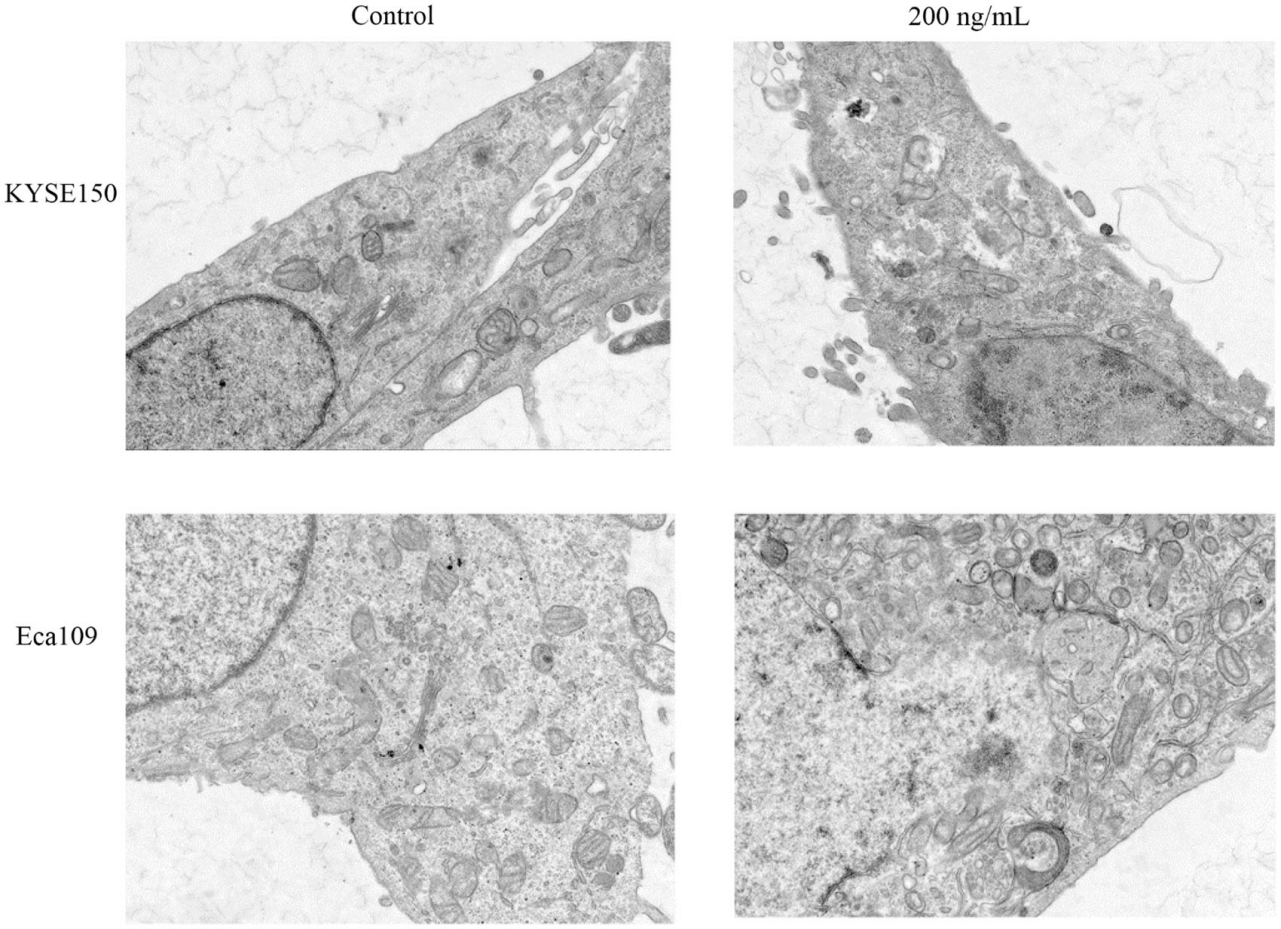
Fv-LDP-D3-AE induced changes in the ultrastructure of esophageal cancer cells.

Transmission electron microscopy was used to study the effect of Fv-LDP-D3-AE on the ultrastructure of EC cells. In Fv-LDP-D3-AE-treated KYSE150 cells, mitochondria were severely swollen, the mitochondrial membranes were severely disintegrated, the matrix was uneven, cristae were broken and reduced, indicative of a prominent mitochondrial damage compared to the control. Similar changes were found in Eca109 cells (Figure 6). Taken together, these results indicated that Fv-LDP-D3-AE can damage the mitochondria of EC cells.

### 3.7. In vivo and in vitro fluorescence imaging of Fv-LDP-D3

**Figure 7.**
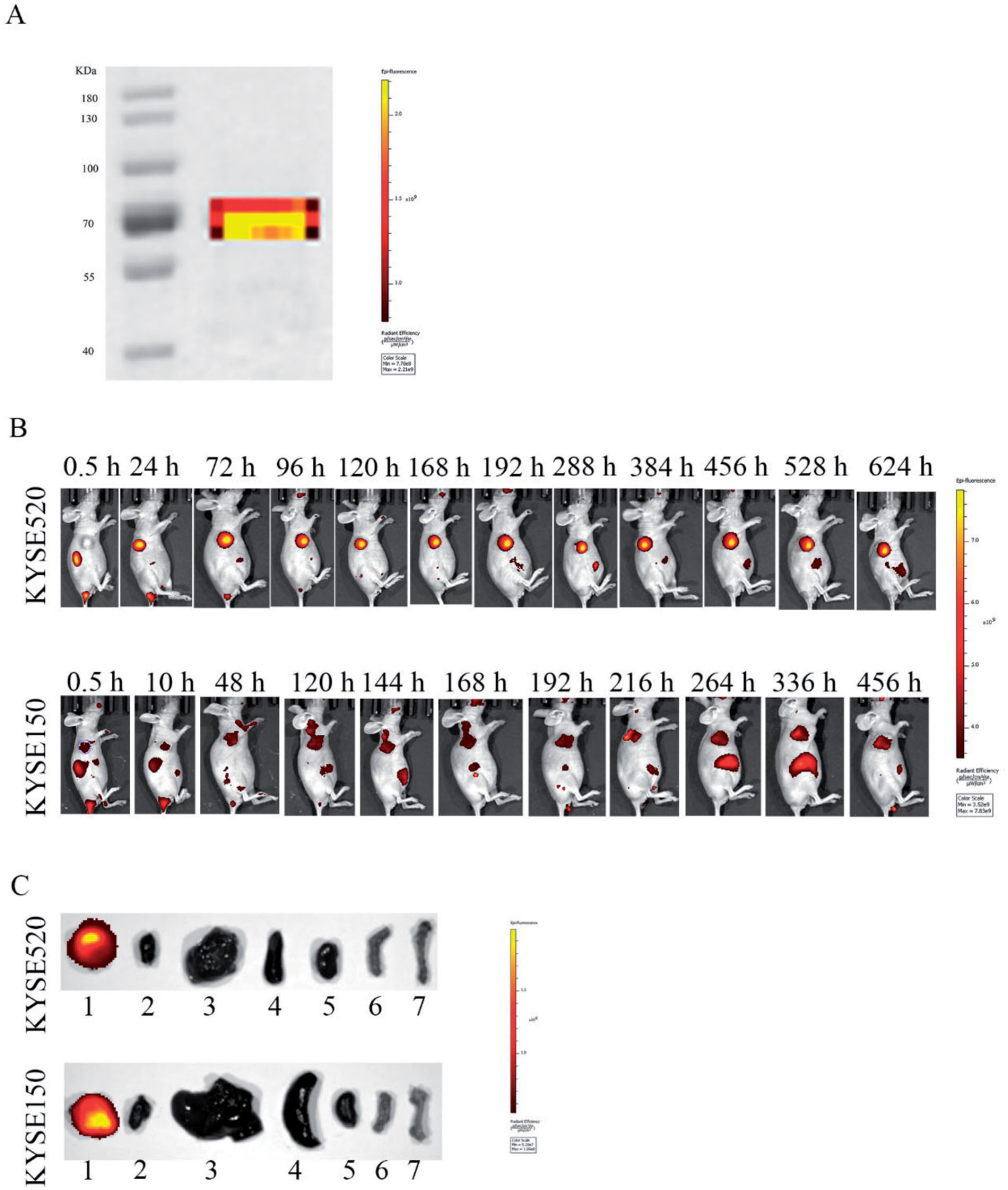
Fluorescence imaging of Dylight680-labeled Fv-LDP-D3 in KYSE520 and KYSE150 xenograft-bearing nude mice. (A) Electrophoresis of DyLight680-labeled Fv-LDP-D3. (B) *In vivo* fluorescence imaging of KYSE520 and KYSE150 xenograft athymic mouse models after injection of 20 mg/kg Fv-LDP-D3 via the tail vein. (C) *In vitro* fluorescence images; 1–7 representing tumor, heart, liver, spleen, kidney, small intestine, and femur taken from the dissected xenograft-bearing mice, respectively.

The tissue distribution and tumor targeted accumulation of Fv-LDP-D3 in KYSE520 and KYSE150 xenograft mouse models were evaluated using an optical molecular imaging system. As shown in Figure 7(A), the fusion protein Fv-LDP-D3 was successfully labeled with DyLight680. Fv-LDP-D3 exhibited superior tumor-targeting capability in KYSE520 xenograft models compared to in KYSE150 xenograft-bearing mice. The fluorescence intensity observed in nude mice subcutaneously inoculated with KYSE520 cells peaked at 72 h after intravenous administration. Notably, *in vivo*, DyLight680-labeled Fv-LDP-D3 targeted the tumor site and the fluorescence persisted for a long-lasting retention over a period of 26 days (Figure 7(B)). Moreover, DyLight680-labeled Fv-LDP-D3 localized in the tumors of nude mice *in vitro*, whereas other organs did not display detectable fluorescence, indicative of Fv-LDP-D3 could be specifically distributed in the tumor site (Figure 7(C)).

### 3.8. In vivo therapeutic efficacy

**Figure 8.**
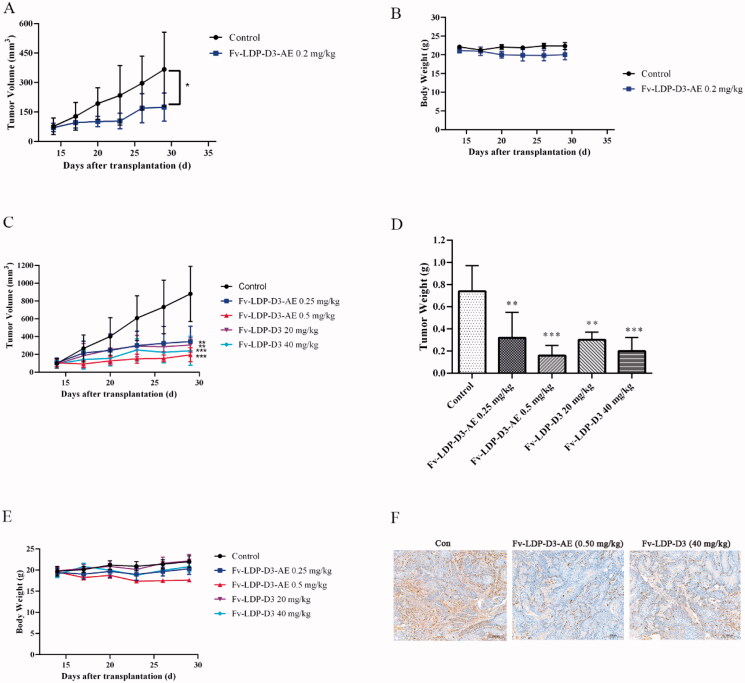
In vivo therapeutic efficacy. (A) Tumor growth curve of the KYSE150 tumor xenograft model (*n* = 6). Mice were treated with Fv-LDP-D3-AE (0.2 mg/kg). (B) Body weight growth curve of ESCC KYSE150 tumor xenograft models. KYSE520 xenograft mice were treated with various doses of Fv-LDP-D3 (20, 40 mg/kg), Fv-LDP-D3-AE (0.25, 0.50 mg/kg). (C) Tumor growth curves. (D) Tumor weights at the end of the experiment. (E) Body weight change curves, and (F) Immunohistochemical detection of Ki-67. *p < 0.05, **p < 0.01, ***p < 0.001 compared with the control.

**Figure 9.**
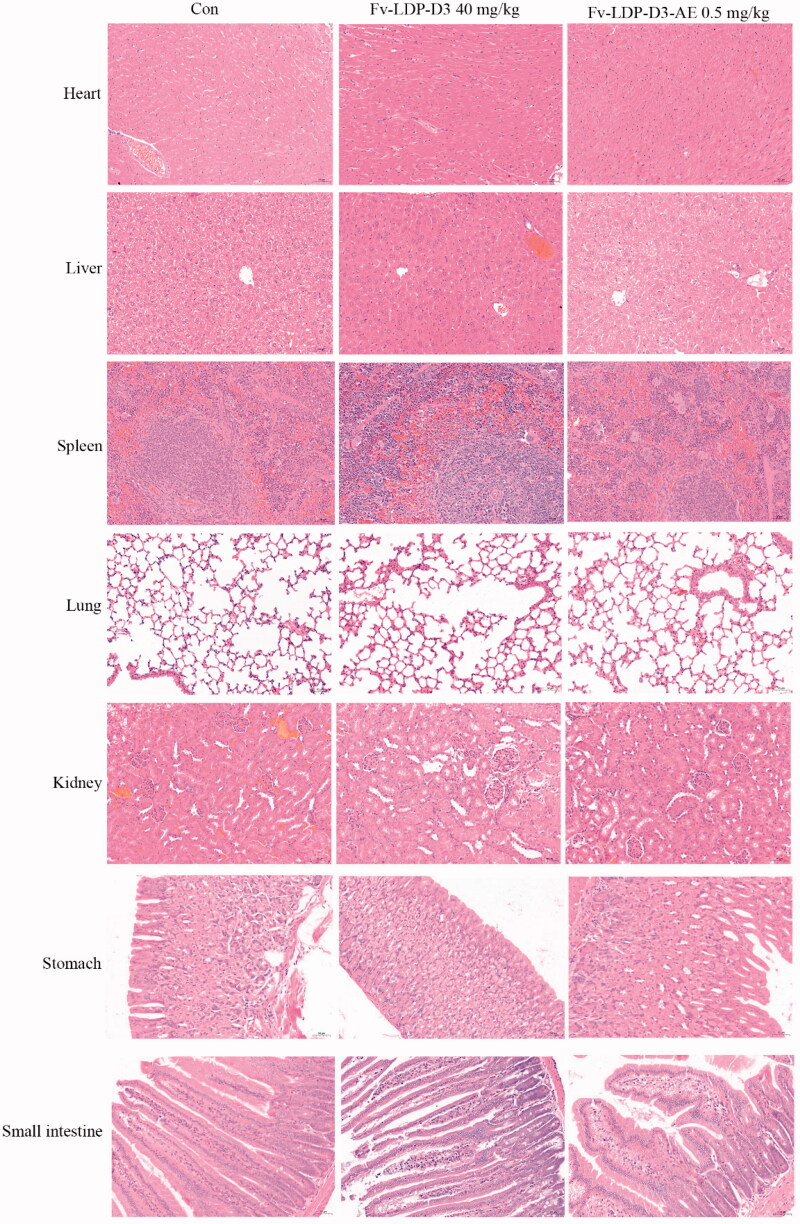
Histopathological examination (H&E staining, ×200) of various organs of the KYSE520 xenograft-bearing athymic mice treated with Fv-LDP-D3 (40 mg/kg), Fv-LDP-D3-AE (0.50 mg/kg), respectively.

We evaluated the therapeutic effect of Fv-LDP-D3-AE against KYSE150 xenograft in athymic mice. During the experimental period, Fv-LDP-D3-AE suppressed tumor growth. The tumor inhibition rate of Fv-LDP-D3-AE at 0.2 mg/kg was 64%, as compared with the controls (*p* < .05) (Figure 8(A)). No prominent toxicity was observed in treated mice as none of them died, and less than 10% weight loss was observed (Figure 8(B)). In order to further study the therapeutic effect of Fv-LDP-D3 and Fv-LDP-D3-AE, we evaluated their anti-tumor activity in the KYSE520 xenograft model. The tumor inhibition rates of Fv-LDP-D3 (20 mg/kg) and Fv-LDP-D3 (40 mg/kg) were 73% and 81%, respectively, and those of Fv-LDP-D3-AE (0.25 mg/kg) and Fv-LDP-D3-AE (0.5 mg/kg) were 69% and 89%, respectively (Figure 8(C,D)). The daily activity and food intake of treated mice were normal, and the observed weight loss was within 10% (Figure 8(E)). Compared with the control group, Fv-LDP-D3 and Fv-LDP-D3-AE inhibited the expression of Ki-67 in tumor tissue (Figure 8(F)). At the end of the experiment, mice were euthanized and specimens of various organs were collected and processed for the preparation of hematoxylin and eosin (H&E) stained sections; tissue was then examined under a microscope. No histopathological changes were found in the heart, liver, spleen, lung, kidney, stomach, and small intestine of mice in the Fv-LDP-D3 (40 mg/kg), and Fv-LDP-D3-AE (0.5 mg/kg) treated groups as well as the controls, indicating that the administered dosage levels were well tolerated (Figure 9).

## 4. Discussion

EC remains associated with high morbidity and mortality rates. While chemotherapy enables the local control of tumor and may prevent distant metastasis, patients often develop drug resistance against chemotherapeutics (Vrana et al., 2018). Although many new therapeutic and diagnostic methods have been introduced, disease recurrence rates remain high, prognosis remains poor, and there still is no curative treatment. In addition, research into the treatment of ESCC has revealed a number of potential therapeutic targets such as orai1-mediated calcium signaling and PFKL (phosphate kinase, live type), however, further research is still needed (Zhu et al., 2014; Cui et al., 2018; Zheng et al., 2022).

EGFR is implicated in tumor development, progression, and metastasis, via downstream signaling cascades involving AKT, MEK, and ERK (Yewale et al., 2013). In our previous study, we constructed a recombinant EGFR/CD13 dual-targeting fusion protein, which exhibited anti-tumor efficacy (Guo et al., 2010; Sheng et al., 2017). Therefore, EGFR-targeting ADC formulations may represent a promising therapeutic approach for the treatment of EC. An albumin-LDM conjugate previously constructed by our research group exhibited anti-tumor efficacy both *in vitro* and *in vivo* (Li et al., 2018). Further, other albumin-based drugs have already been approved for the treatment of cancer (Kratz, 2008). Taken together, albumin is an effective carrier for the specific delivery of drug molecules to tumor sites.

In our previous research, a recombinant fusion protein and ADC based on the third domain of albumin and an anti-EGFR scFv were prepared via recombination technology. In the present study, we demonstrated that the recombinant fusion protein Fv-LDP-D3 has high affinity for EGFR-overexpressing EC cells, ensuring localization to the tumor and reducing toxicity to normal cells. NIH3T3 cells lack EGFR overexpression (Guo et al., 2014), and Fv-LDP-D3 has a very weak affinity for NIH3T3 cells. Fv-LDP-D3 was taken up by EC cells through macropinocytosis. *In vivo* imaging studies demonstrated that Fv-LDP-D3 could selectively accumulate at tumor sites in the KYSE520 xenograft model where it was retained for a long period of time, suggesting that the properties of human serum albumin may prolong the half-life of drugs. We observed superior tumor targeting in the KYSE520 xenograft model compared to the KYSE150 xenograft model, which may be due to the high expression of EGFR in KYSE520 cells. The present findings support the feasibility of using EGFR as the therapeutic molecular target and albumin as the carrier for cancer targeted therapy. Fv-LDP-D3 inhibited the proliferation of EC cells at relatively high concentrations, which was attributed to the suppression of EGFR phosphorylation and IMPDH2 signaling. IMPDH2 is overexpressed in a variety of tumors and can promote cell invasion as well as migration (Duan et al., 2018). Thus, IMPDH2 overexpression is closely related to tumor progression (Zou et al., 2015). Shikonin, a selective IMPDH2 inhibitor, was reported to suppress the growth of triple-negative breast cancer cell line MDA-MB-231 (Wang et al., 2021). The recombinant fusion protein Fv-LDP-D3 suppressed IMPDH2, which provides a new idea for the development of esophageal cancer drugs. Of course, it should be noted that targeting inhibition of p-EGFR/IMPDH2 signaling may be able to improve esophageal cancer treatment efficacy, and the question as to whether there is a relationship between IMPDH2 and EGFR signaling requires in-depth experimental exploration.

Fv-LDP-D3 induced the apoptosis of EC cells *in vitro* by upregulating apoptotic factors (including cleaved PARP, cleaved caspase-3) and suppressing the expression of anti-apoptotic proteins (such as Bcl-2). Various anti-cancer drugs cause DNA damage, leading to the activation of cell cycle checkpoints and subsequent proliferation arrest (Montano et al., 2012). Fv-LDP-D3-AE treatment induced DNA damage, as indicated by the increased expression of p-Histone H2A.X, while suppressing Chk1 expression. In addition, Fv-LDP-D3-AE inhibited the migration and invasion of EC cells *in vitro*, induced apoptosis, induced cycle arrest at G2/M, and compromised mitochondrial structure. Fv-LDP-D3-AE also suppressed EGFR phosphorylation. Further, Fv-LDP-D3-AE (0.2 mg/kg) exhibited a tumor inhibition rate of 64% in KYSE150 mouse xenograft model. Additionally, in the KYSE520 tumor model, both the recombinant fusion protein Fv-LDP-D3 and its antibody–drug conjugate have shown strong therapeutic effects. In particular, the antibody-drug conjugate has improved therapeutic efficacy compared to recombinant fusion proteins. Taken together, our research suggests that Fv-LDP-D3 and Fv-LDP-D3-AE may be promising agents for the treatment of EGFR-positive esophageal tumors.

## 5. Conclusions

In summary, the present study demonstrated that the recombinant fusion protein Fv-LDP-D3 which consists of the Fv fragment of an anti-EGFR antibody, the apoprotein of lidamycin (LDP), and the third domain of human serum albumin (D3) and its pertinent drug conjugate (Fv-LDP-D3-AE) which is prepared by integrating the active enediyne chromophore (AE) into the fusion protein molecule both exhibited strong anti-tumor activity against EC cells *in vitro* and effectively inhibited the growth of cancer xenografts *in vivo*. The current findings highlight the therapeutic potential of Fv-LDP-D3 and Fv-LDP-D3-AE for esophageal cancer.
